# Quantification of localized NAD^+^ changes reveals unique specificity of NAD^+^ regulation in the hypothalamus

**DOI:** 10.1038/s41514-023-00098-1

**Published:** 2023-01-25

**Authors:** Sean Johnson, Kiyoshi Yoshioka, Cynthia S. Brace, Shin-ichiro Imai

**Affiliations:** 1 Department of Gerontology, Laboratory of Molecular Life Science, Institute of Biomedical Research and Innovation, Kobe, Japan; 2 Institute for Research on Productive Aging (IRPA), Tokyo, Japan; 3 Department of Developmental Biology, Washington University School of Medicine, St. Louis, MO 63110 USA

**Keywords:** Mouse, Ageing

## Abstract

Recently, it has become a consensus that systemic decreases in NAD^+^ are a critical trigger for age-associated functional decline in multiple tissues and organs. The hypothalamus, which contains several functionally distinct subregions called nuclei, functions as a high-order control center of aging in mammals. However, due to a technical difficulty, how NAD^+^ levels change locally in each hypothalamic nucleus during aging remains uninvestigated. We were able to establish a new combinatorial methodology, using laser-captured microdissection (LCM) and high-performance liquid chromatography (HPLC), to accurately measure NAD^+^ levels in small tissue samples. We applied this methodology to examine local NAD^+^ changes in hypothalamic nuclei and found that NAD^+^ levels were decreased significantly in the arcuate nucleus (ARC), ventromedial hypothalamus (VMH), and lateral hypothalamus (LH), but not in the dorsomedial hypothalamus (DMH) of 22-month-old mice, compared to those of 3-month-old mice. The administration of nicotinamide mononucleotide (NMN) significantly increased NAD^+^ levels in all these hypothalamic nuclei. Interestingly, the administration of extracellular nicotinamide phosphoribosyltransferase-containing extracellular vesicles (eNampt-EVs) purified from young mice increased NAD^+^ levels in the ARC and DMH. These results reveal the unique specificity of NAD^+^ regulation in the hypothalamus during aging.

## Introduction

Recently, decreases in tissue NAD^+^ levels have been demonstrated to play an important, universal role in triggering age-associated physiological decline at a systemic level^1–5^. Indeed, it has been reported that NAD^+^ levels decrease with age in many different tissues and organs, including adipose tissue^6,7^, skeletal muscle^6–10^, liver^6,7,10–12^, pancreas^7^, skin^13^, retina^14^, and different regions of the brain^15–17^. For example, hippocampal NAD^+^ levels decline with age, contributing to age-associated depletion of the neural stem/progenitor cell pool^17^ and age-related changes in sensory processing and emotionality, termed cognitive hypersensitivity^15^. In particular, genetic ablation of nicotinamide phosphoribosyltransferase (Nampt), a key rate-limiting NAD^+^ biosynthetic enzyme in mammals, in the CA1 region, but not in the dentate gyrus, recapitulates the age-associated cognitive hypersensitivity phenotype even in young mice, revealing a necessity of measuring local NAD^+^ changes within a tissue^15^. Additionally, pathological conditions such as diabetes-induced memory deficits and Alzheimer’s disease exhibit significant decreases in NAD^+^ levels in the brain, and NAD^+^ boosting by NAD^+^ intermediates can ameliorate these symptoms^18–20^. Nonetheless, it is still a technical challenge to accurately measure NAD^+^ levels within an organ or tissue. We have previously established a methodology that allows accurate measurement of NAD^+^ with high-performance liquid chromatography (HPLC)^21^. The key in this methodology is the use of a very strong acid, perchloric acid (HClO_4_), to extract NAD^+^ from tissues with a minimal loss of NAD^+^. This particular extraction process becomes more important when we have to deal with small amounts of tissue samples. To examine local NAD^+^ changes in a small region within a tissue, we decided to employ laser-captured microdissection (LCM) and then immediately process such a small sample with HClO_4_, allowing us to measure local NAD^+^ changes accurately.

The hypothalamus has been demonstrated to function as a high-order control center of aging in mammals^22,23^. Our previous study has demonstrated that two subregions within the hypothalamus, namely the dorsomedial and lateral hypothalamic nuclei (DMH and LH, respectively), play a critical role in the regulation of mammalian aging and longevity^22^. In the DMH and LH, Sirt1, a mammalian NAD^+^-dependent protein deacetylase, cooperates with Nkx2-1, a homeodomain transcription factor, and regulates the expression of key genes, including *orexin type 2 receptor* (*Ox2r*) and *PR domain containing 13* (*Prdm13*), promoting neuronal activity to delay aging and extend lifespan in mice^22^. It has been speculated that Sirt1 activity declines over age due to the decrease in NAD^+^ within these regions^24,25^. However, due to a technical difficulty, whether NAD^+^ levels decrease in each hypothalamic nucleus has never been investigated. In our present study, we addressed this question by using a new combinatorial methodology that we developed.

## Results

### Quantification of NAD^+^ levels in each hypothalamic nucleus

**Fig. 1:**
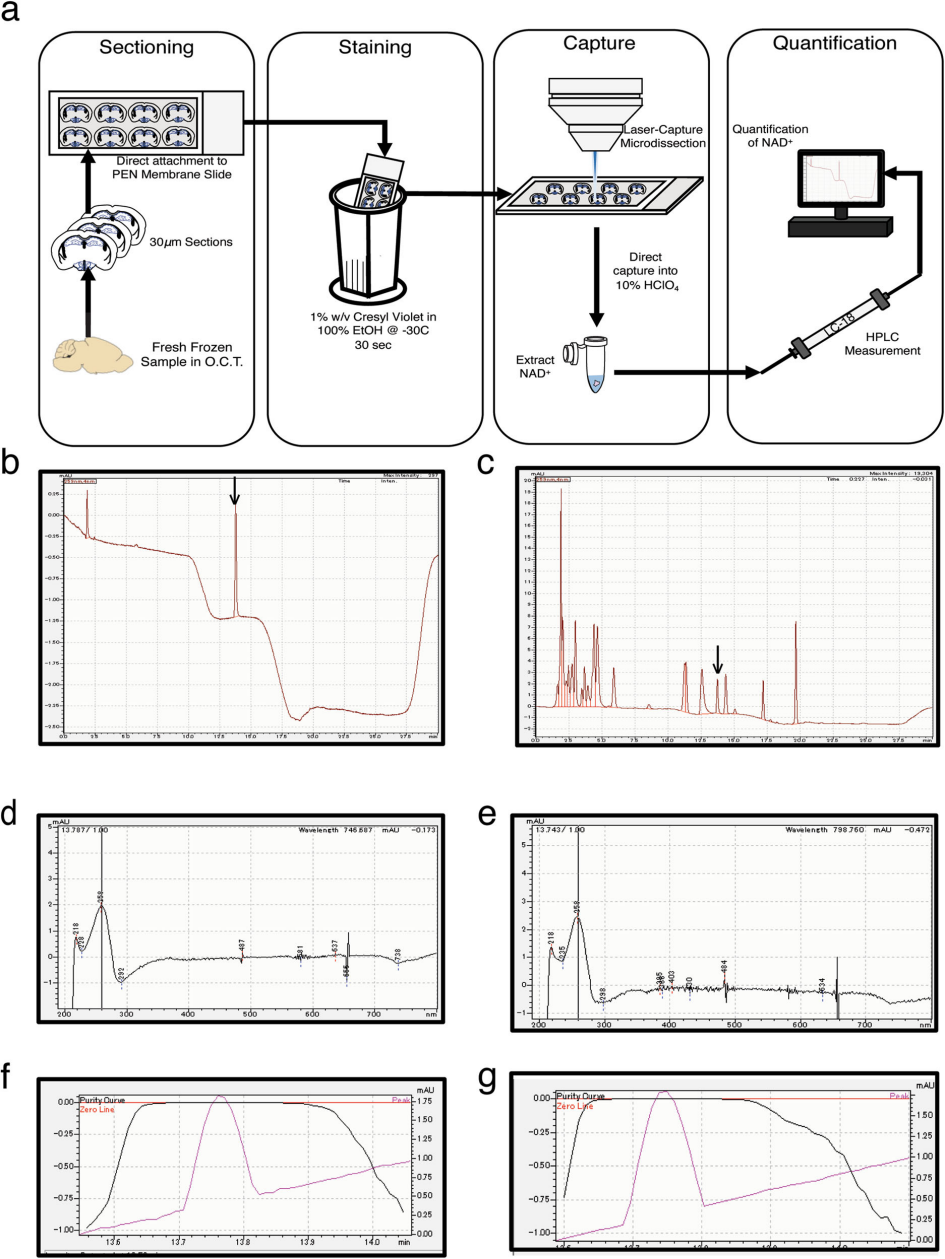
A new combinatorial methodology of NAD^+^ measurement using laser-captured microdissection (LCM) and high-performance liquid chromatography (HPLC). **a** A scheme showing the methodology. **b**–**g** Chromatograms measured at 259 nm of standard NAD^+^ (**b**) and NAD^+^ in LCM-collected DMH (**c**) are shown. Absorbance spectra (**d**, **e**) and purity measurements (**f**, **g**) of peaks of standard NAD^+^ (**d**, **f**) and NAD^+^ in LCM-collected DMH (**e**, **g**) are also presented. NAD^+^ peaks, identified by arrows (**b**, **c**), were eluted at 13.7 min.

We attempted to measure local NAD^+^ levels in each hypothalamic nucleus by employing LCM to collect small tissue samples. Careful optimization was required to conduct LCM with minimal loss of NAD^+^. The entire scheme of this methodology is shown in Fig. 1a. Briefly, freshly frozen brain samples were prepared with the OCT compound, and 30 μm sections were cut, spanning the hypothalamus from Bregma −1.8 to −2.0 mm. The brain sections were directly mounted to PEN-membrane slides. When samples were processed for LCM collection, the sections were stained for 30 s in a 1% Cresyl Violet w/v 100% ethanol solution kept at –30 °C. At this step, care should be given to minimize sample exposure to room temperature until dehydrated to avoid degradation of NAD^+^. The LCM was performed to collect the arcuate nucleus (ARC), ventromedial hypothalamic nucleus (VMH), DMH, and LH. Samples were collected directly into a 10% HClO_4_ solution. One sample should be processed at a time to avoid extended exposure to room temperature. After sample collection with LCM, NAD^+^ was extracted, as previously described^21^.

With this methodology, NAD^+^ was reliably detected in each hypothalamic nucleus (Fig. 1c, e, g). The elution times, spectrogram patterns, and purity curves were consistent with those of standard NAD^+^ (Fig. 1a, d, f). The clear detection of NAD^+^ in such a small sample gave support and allows us to measure local NAD^+^ levels in a variety of physiological and therapeutic conditions.

### NAD^+^ decreases with age in the ARC, VMH, and LH, but not in the DMH

**Fig. 2:**
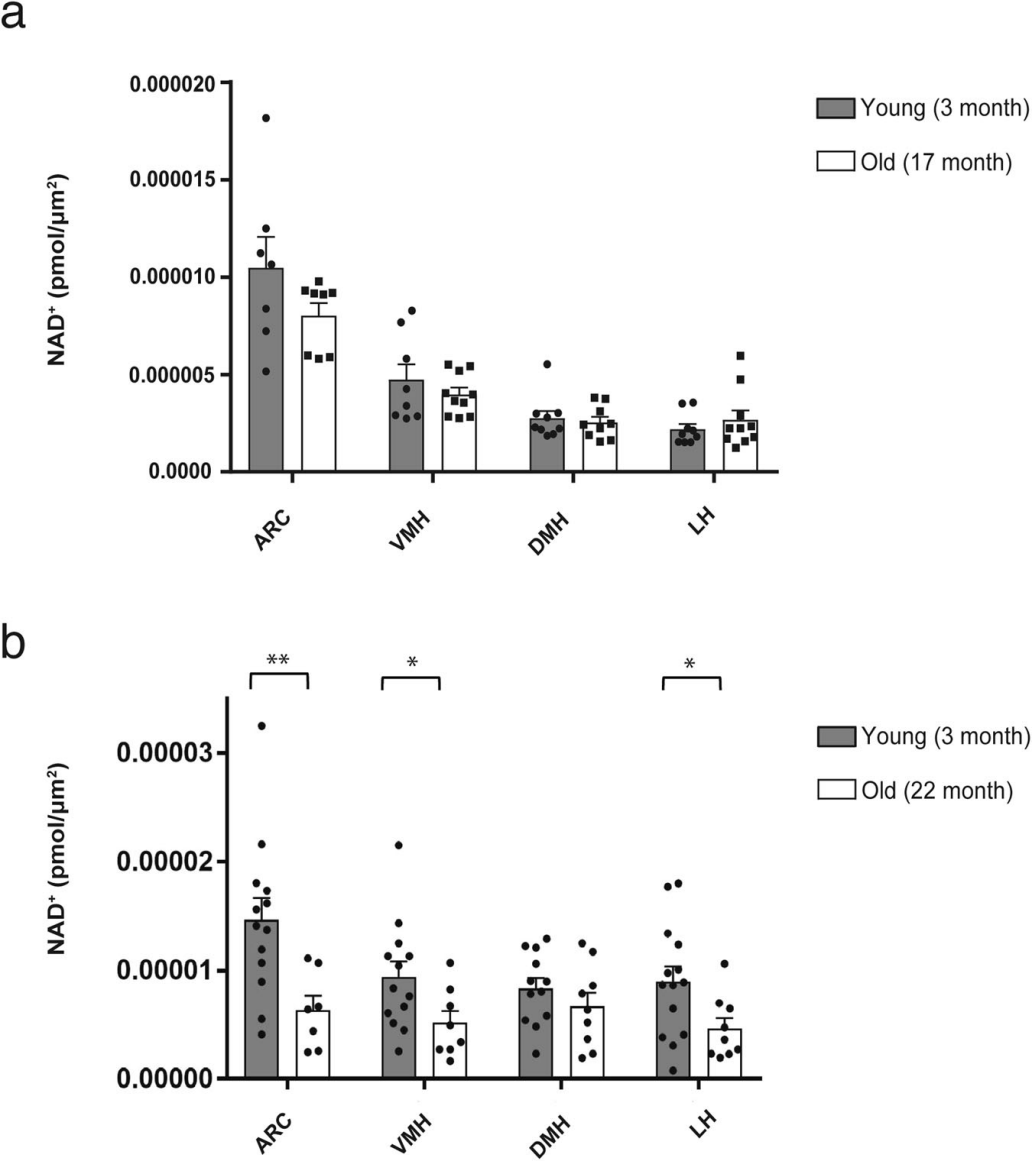
Local NAD^+^ levels in each hypothalamic nucleus do not change at 17 months of age, but decrease significantly in the ARC, VMH, and LH, but not in the DMH, by 22 months of age. **a** NAD^+^ levels in the ARC, VMH, DMH, and LH of male mice at 3 and 17 months of age are shown (*n* = 7–9). **b** NAD^+^ levels in the ARC, VMH, DMH, and LH of male mice at 3 and 22 months of age are shown (*n* = 7–14) **p ≤ 0.01 and *p ≤ 0.05 by unpaired Student’s *t*-test. Error bars indicate s.e.m. ARC arcuate nucleus, VMH ventromedial hypothalamic nucleus, DMH dorsomedial hypothalamic nucleus, LH lateral hypothalamic nucleus.

We first measured NAD^+^ levels in the ARC, VMH, DMH, and LH in wild-type B6 mice at 3 and 17 months of age (Fig. 2a). No significant differences in NAD^+^ levels were observed at this time point. NAD^+^ levels in hypothalamic nuclei from 3-month-old mice tended to be varied more than those in 17-month-old mice. On the other hand, when comparing NAD^+^ levels at 3 and 22 months of age, significant decreases were detected in the ARC, VMH, and LH, but not in the DMH (Fig. 2b). Whereas NAD^+^ levels were again varied more in young hypothalamic nuclei, NAD^+^ in the ARC, VMH, DMH, and LH at 22 months of age showed similar levels, a different trend from 17 months of age. It should be noted that NAD^+^ levels tend to be preserved in the DMH at a later stage of life, compared to other hypothalamic nuclei.

### NAD^+^ levels are restored by the supplementation of nicotinamide mononucleotide (NMN)

**Fig. 3:**
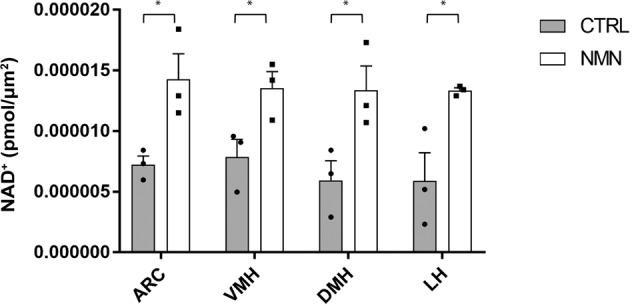
A single intraperitoneal injection of nicotinamide mononucleotide (NMN) significantly increases NAD^+^ levels in all hypothalamic nuclei. NAD^+^ levels in the ARC, VMH, DMH, and LH are shown in male mice at 22 months of age at 30 min after NMN administration at the dose of 300 mg/kg NMN (*n* = 3). Control, age-matched mice were injected with PBS. *p ≤ 0.05 by unpaired Student’s *t*-test. Error bars indicate s.e.m.

NMN supplementation is an effective intervention that can increase the levels of NAD^+^ in many tissues^5^. As previously reported, a single intraperitoneal (IP) injection of NMN at a dose of 500 mg/kg body weight increased hepatic and hippocampal NAD^+^ levels significantly within 15 min^7,17^. Interestingly, single IP injection of NMN at a dose of 300 mg/kg body weight significantly increased NAD^+^ levels in all ARC, VMH, DMH, and LH of 22-month-old mice, compared to PBS-injected age-matched control mice, at 30 min time point after NMN injection (Fig. 3). This finding further confirms that this combinatorial technique of LCM and HPLC is capable of measuring local NAD^+^ changes caused by an NAD^+^-boosting intervention. Additionally, these hypothalamic nuclei of aged mice are still able to synthesize relatively high levels of NAD^+^ as far as they can get access to enough NMN.

### The mRNA expression of NAD^+^ biosynthetic enzymes are unchanged

**Fig. 4:**
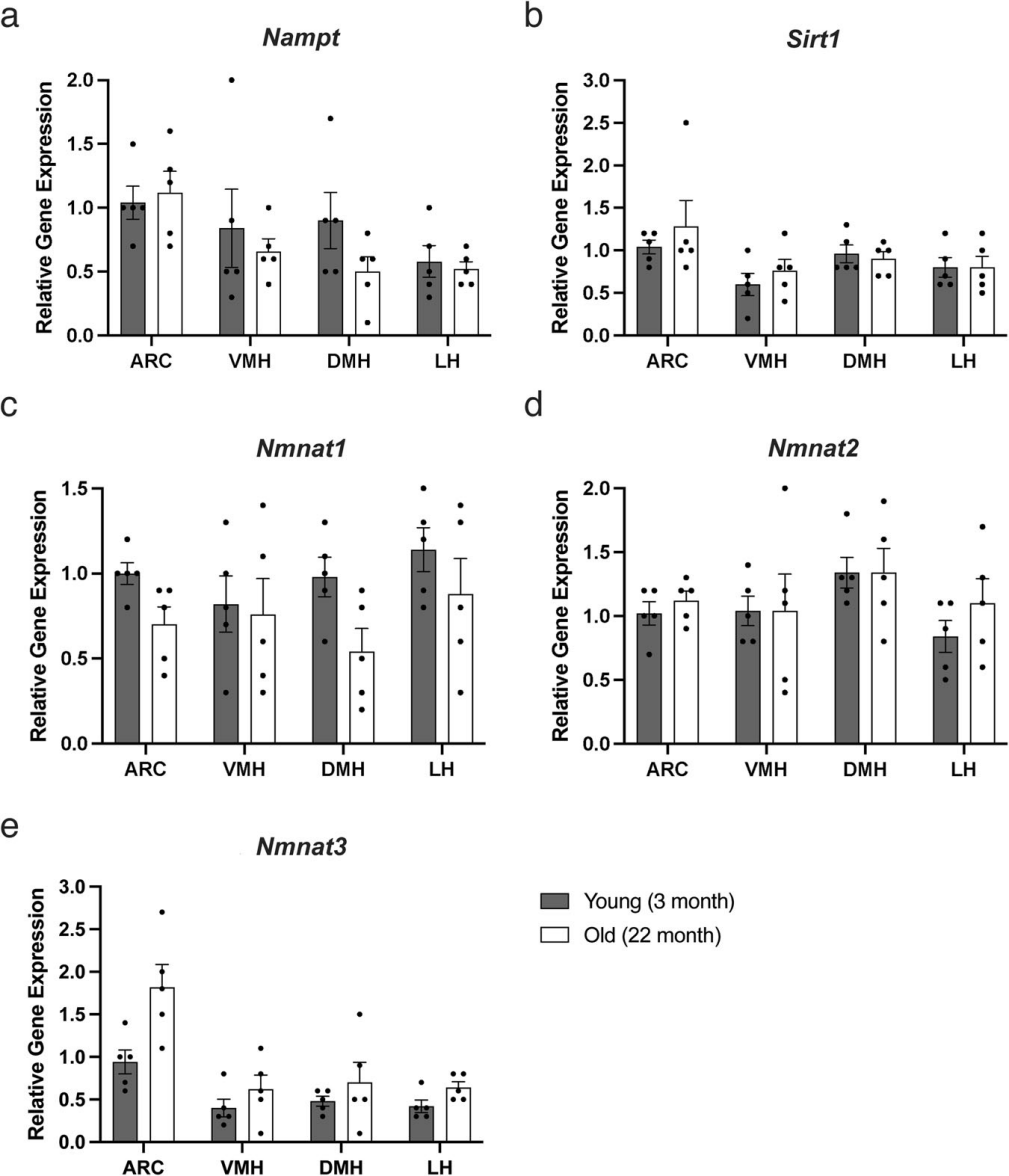
The mRNA expression levels of NAD^+^ biosynthetic genes and *Sirt1* are not altered in aged mice. The mRNA levels of *Nampt* (**a**), *Sirt1* (**b**), *Nmnat1-3* (**c**–**e**) in the ARC, VMH, DMH, and LH of young (3 months) and old (22 months) mice are shown (*n* = 5). The expression levels of each gene are normalized to those in the ARC of young mice. Error bars indicate s.e.m.

Next, we examined whether mRNA expression levels of NAD^+^ biosynthetic enzymes, including *Nampt* and *Nmnat1/2/3* and a mammalian sirtuin *Sirt1,* were changed in the hypothalamic nuclei between 3 and 22 months of age (Fig. 4). Although individual variations were relatively large, the mRNA expression levels of these NAD^+^ biosynthetic enzymes were not significantly altered, suggesting that the main NAD^+^ biosynthetic pathway in the hypothalamus remained robust with age and was capable of synthesizing NAD^+^.

### DNA damage-responding and cytokine genes are upregulated in aged hypothalamic nuclei

**Fig. 5:**
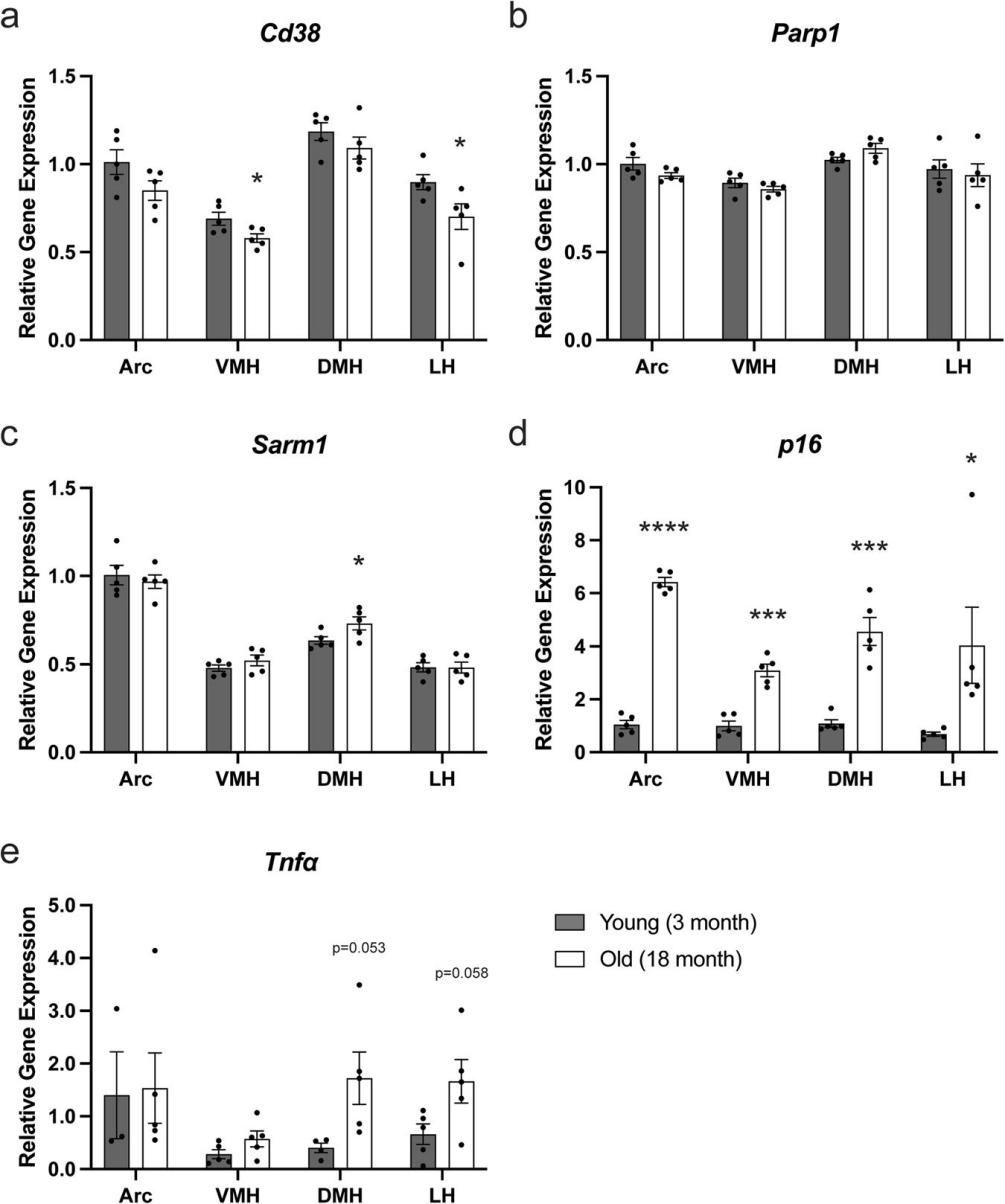
THe mRNA expression levels of DNA damage response and cytokine genes are upregulated in aged hypothalamic nuclei. The mRNA expression levels of genes associated with NAD^+^ consumption (*Cd38*, *Parp1*, and *Sarm1*; **a**–**c**), DNA damage (*p16*; **d**), and inflammation (*Tnfα*; **e**) in the ARC, VMH, DMH, and LH of young (3 months) and old (18 months) mice are shown (*n* = 3–5). The expression levels of each gene are normalized to those in the ARC of young mice. *p ≤ 0.05 **p ≤ 0.01 ***p ≤ 0.001 ****p ≤ 0.0001 by unpaired Student’s *t*-test. Error bars indicate s.e.m.

Because we did not detect any significant differences in mRNA expression in the main NAD^+^ biosynthetic genes and *Sirt1*, we decided to compare mRNA expression of genes associated with NAD^+^ consumption (*Cd38*, *Parp1*, and *Sarm1*), DNA damage (*p16*), and inflammation (*Tnfα*) between young and aged hypothalamic nuclei. Whereas *Cd38* expression tends to show slight decreases throughout all aged hypothalamic nuclei (Fig. 5a), *Parp1* and *Sarm1* expression did not differ between young and aged hypothalamic nuclei (Fig. 5b, c). Interestingly, *p16* expression was significantly upregulated in all aged hypothalamic nuclei, indicating that DNA damages were accumulated in aged hypothalamic nuclei (Fig. 5d). Additionally, *Tnfα* expression also tends to be upregulated in the VMH, DMH, and LH, but not in the ARC, although the absolute expression levels of *Tnfα* were still very low (Fig. 5e). These results suggest that some inflammatory reaction might be triggered in the VMH, DMH, and LH. It has been known that DNA damages and inflammatory cytokines, particularly Tnfα, induce the reduction in NAD^+^ levels^7,26,27^. Thus, these changes might explain NAD^+^ decreases in aged hypothalamic nuclei.

### eNampt-EVs purified from young mice increase NAD^+^ levels in the ARC and DMH

**Fig. 6:**
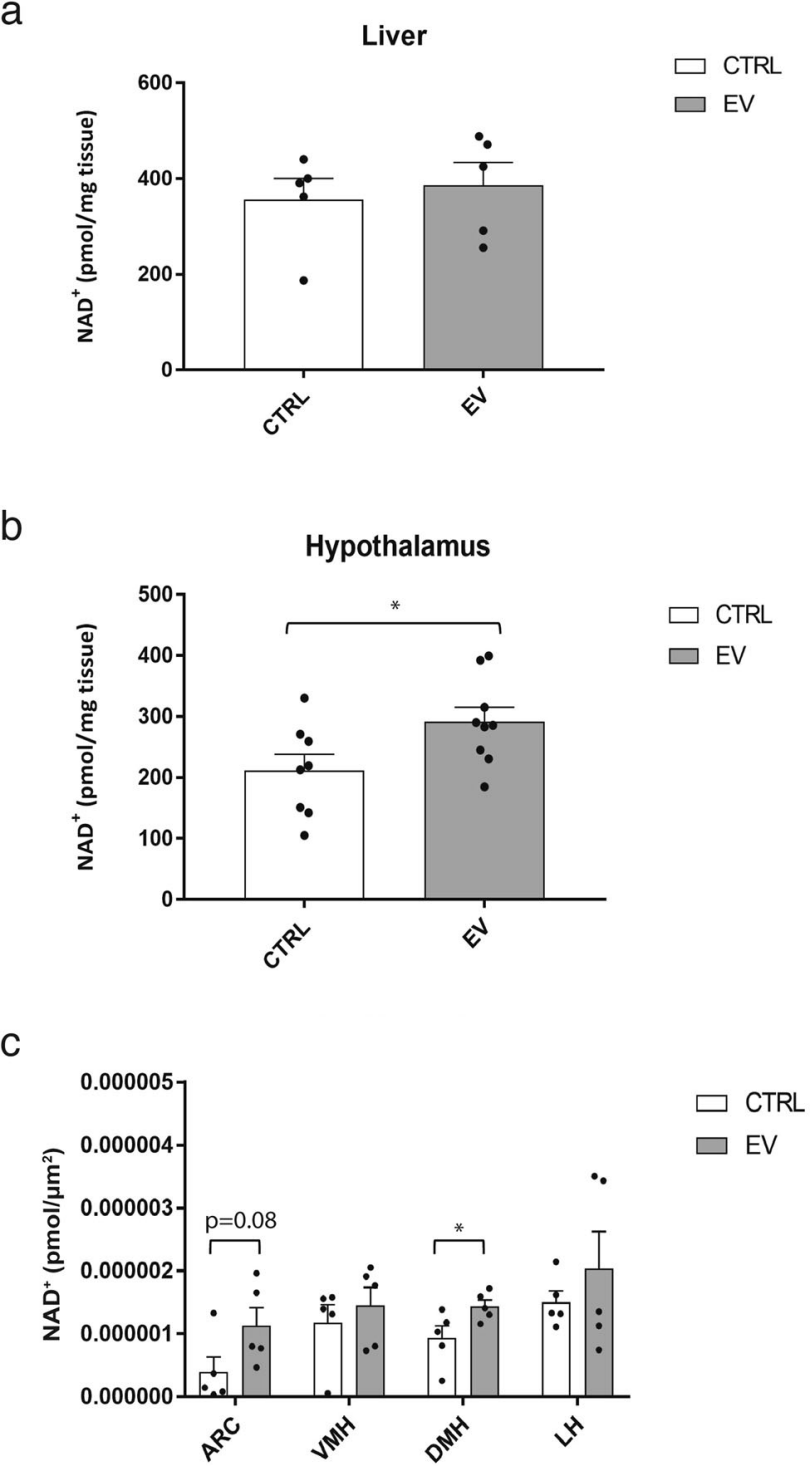
eNampt-EVs purified from young mice boost NAD^+^ in the hypothalamus, particularly in the ARC and DMH, but not the liver. **a** NAD^+^ levels in liver samples of 22-month-old mice 30 min after administration of eNampt-EVs or PBS (*n* = 5). **b** NAD^+^ levels in the whole hypothalami of 22-month-old mice 30 min after administration of eNampt-EVs or PBS (*n* = 8). **c** NAD^+^ levels in the ARC, VMH, DMH, and LH of 22-month-old mice 30 min after administration of eNampt-EVs or PBS (*n* = 5). *p ≤ 0.05 by unpaired Student’s *t*-test. Error bars indicate s.e.m.

It has been reported that hypothalamic NAD^+^ levels are regulated by extracellular Nampt (eNampt) that is encapsulated into small extracellular vesicles (eNampt-EVs) and secreted from adipose tissue into the blood circulation in both mice and humans^28,29^. Circulating eNampt levels have also been shown to decrease over age in both mice and humans^29^. Therefore, we speculated that decreased eNampt might cause observed decreases in hypothalamic NAD^+^ levels. To address this hypothesis, we purified eNampt-EVs from young mice, administered them to 22-month-old mice intravenously, and measured whole NAD^+^ levels in the liver and hypothalamus. Whereas no significant increases in NAD^+^ levels were detected in the liver (Fig. 6a), NAD^+^ levels were significantly increased in the whole hypothalami (Fig. 6b), implicating tissue specificity for eNampt-EVs. Furthermore, we measured local NAD^+^ changes in each hypothalamic nucleus after intravenous (IV) injection of purified eNampt-EVs (Fig. 6c). Interestingly, only the DMH and ARC showed clear increases in NAD^+^ levels after eNampt-EV injection. These findings suggest that the hypothalamus has unique requirements for NAD^+^ biosynthesis and may rely on an external source of Nampt, namely, eNampt-EVs, in blood circulation.

## Discussion

Whereas more lines of evidence have been accumulating for the importance of systemic NAD^+^ decreases in the pathogenesis of age-associated functional decline^1–5^, our need to measure local NAD^+^ changes within a tissue or an organ has also been increasing. Even when a mass spectrometry-driven methodology is available these days, there has been a technical challenge to allow us to accurately measure local NAD^+^ levels in small tissue samples. Such a challenge particularly stands out for the hypothalamus, that is divided into several functionally distinct subregions called nuclei. In our present study, we successfully established a new combinatorial methodology that employed LCM and HPLC to measure local NAD^+^ levels in each hypothalamic nucleus. By using this new methodology, we demonstrated that NAD^+^ levels were decreased locally in the ARC, VMH, and LH of 22-month-old mice, compared to those of 3-month-old mice. Interestingly, NAD^+^ in the DMH were preserved even at 22 months of age. All these hypothalamic nuclei showed significant increases in NAD^+^ levels in response to NMN administration. Additionally, while the mRNA expression levels of major NAD^+^ biosynthetic enzymes and *Sirt1* were unchanged in aged hypothalamic nuclei, *p16* and *Tnfα* expression levels were upregulated in VMH, DMH, and LH, suggesting that DNA damage and inflammation may contribute to NAD^+^ reduction in these hypothalamic nuclei. Because it has been shown that circulating eNampt levels, which control hypothalamic NAD^+^ levels^28^, decrease over age in both mice and humans^29^, we administered eNampt-EVs purified from young mice to aged mice and confirmed the NAD^+^ increase in the whole hypothalamus. Our newly established methodology further revealed that the DMH and ARC responded to eNampt-EV administration and increased their NAD^+^ levels. These results clearly demonstrated the effectiveness of the combinatorial methodology that we developed in this study for the accurate measurement of local NAD^+^ changes in small samples, such as hypothalamic nuclei.

**Fig. 7:**
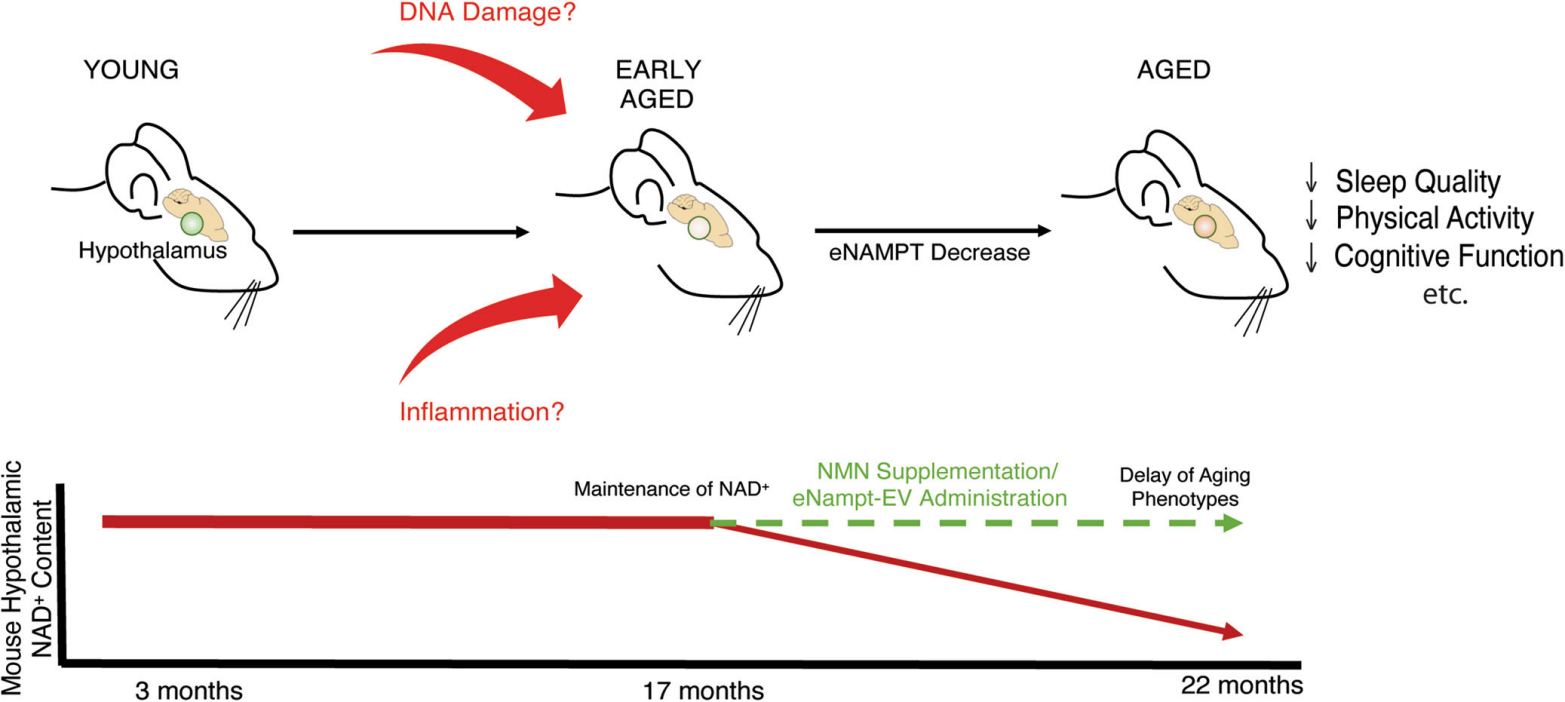
A model for how hypothalamic NAD^+^ changes during aging and potential therapeutic interventions to ameliorate age-associated functional decline. Briefly, as mice age, the level of NAD^+^ in the hypothalamus decreases due to various factors, including DNA damage and inflammation, correlating to age-related behavioral phenotypes. NMN and/or eNampt-EV administration can act as NAD^+^-boosting interventions delaying the onset and progression of age-related phenotypes.

It should be noted that local NAD^+^ levels in hypothalamic nuclei are well maintained even at 17 months of age, a distinct feature compared to other peripheral tissues and brain regions. In the hippocampus, for example, both *Nampt* mRNA and Nampt protein levels show significant decreases at 18–20 months of age, and NAD^+^ levels also decrease accordingly^15,17^. Because we detected no significant changes in *Nampt* mRNA levels in aged hypothalamic nuclei, it is likely that Nampt protein levels would also show no change over age in these hypothalamic nuclei. These findings strengthen the importance of NAD^+^ maintenance in the hypothalamus, that functions as a high-order control center of aging. It seems that the resources necessary to maintain NAD^+^ are allocated for the hypothalamus to maintain its function. This may particularly be the case for the DMH because local NAD^+^ levels in the DMH were still preserved at 22 months of age. eNampt-EVs may contribute to this NAD^+^ preservation in the DMH. Indeed, we have previously demonstrated that genetic or pharmacological supplementation of eNampt-EVs can improve sleep quality and wheel-running activity in aged mice^29^. With these results, we propose a model for hypothalamic NAD^+^ changes during aging, highlighting the maintenance of NAD^+^, and the potential of NAD^+^-boosting anti-aging interventions, to ameliorate age-associated functional decline (Fig. 7).

How can the hypothalamus maintain its NAD^+^ levels? The rate-limiting step in the major NAD^+^ biosynthetic pathway is the conversion of nicotinamide to NMN by Nampt^30–32^. Previous studies have shown that each tissue has different levels of Nampt^33^, and the brain in general and the hypothalamus have comparatively low levels of Nampt, implying that the hypothalamus may utilize an additional pathway to maintain the synthesis of NMN^28,29^. Indeed, it is eNampt-EVs, actively secreted from adipose tissue, that regulates hypothalamic NAD^+^ levels remotely^28,29^. Increasing circulating eNampt-EV levels in aged mice significantly increases NAD^+^ levels in multiple tissues, including the hypothalamus, hippocampus, pancreas, and retina^29^. Our results demonstrated that supplementation of eNampt-EVs purified from young mice significantly increased the NAD^+^ levels in the whole hypothalamus, the DMH and ARC in particular, in aged mice. Intriguingly, the NAD^+^ levels in the liver were not changed in response to eNampt-EV administration, consistent with our previous result^29^, revealing an interesting tissue specificity of eNampt-EVs. Whereas the mechanism of this tissue specificity for eNampt-EVs currently remains unknown, these findings strongly suggest that the supplementation of eNampt-EVs is an effective therapeutic intervention to boost NAD^+^ biosynthesis in a tissue-specific manner. NMN supplementation was also proven to increase NAD^+^ in all hypothalamic nuclei, as well as other peripheral tissues, as previously reported^4,5^. It will be of great interest to investigate the molecular basis for differences in the NAD^+^-boosting actions of eNampt-EVs and NMN.

In conclusion, we established a new combinatorial methodology to extend the spatial resolution of NAD^+^ measurement. This methodology can be used to monitor NAD^+^ changes in each hypothalamic nucleus to better understand the differential regulation of NAD^+^ biosynthesis in the hypothalamus that plays a key role in mammalian aging and longevity control. This methodology can also be applied to pathophysiologies which may affect NAD^+^ levels in other brain regions and peripheral tissues, potentially showing new targets for NAD^+^-boosting interventions. Given the interesting tissue specificity of eNampt-EVs, the selective increase in NAD^+^ by using eNampt-EVs will provide an additional tool to dissect the importance of NAD^+^ biosynthesis in aging and the development of another effective anti-aging intervention.

## Methods

### Tissue preparation, sectioning, and laser-captured microdissection

#### Tissue sample preparation

Mice were sacrificed by cervical dislocation, and brains were removed and placed into the brain matrix (Kent Scientific, RBMA-200C). Unnecessary tissues were carefully removed with single-blade razors. Brains were then placed into Cryomold No.2 (Tissue-Tek® Cryomold®, 4566). Brain samples were coated with 1:2 20% Sucrose/O.C.T. and frozen on dry ice. Once OCT turned solid white, brain samples could be stored at −80 °C.

#### Cryosectioning

OCT-embedded brain samples were mounted to a specimen stage of a cryostat (CM1950, Leica Biosystems). Excess OCT was carefully removed by sectioning and then a single-edged razor. Samples were then cut at a thickness of 30 μm. Eight sections were directly mounted using brushes to the central area of a PEN-membrane slide which was kept at room temperature (Carl Zeiss, 415190-9041-000). Samples were kept in the cryostat until the completion of the sampling procedure. Once all samples were collected, they were placed in pre-chilled small-size slide boxes, wrap slide boxes with parafilm, and store at −80 °C.

#### Staining and dehydration

These two processes were combined to avoid NAD^+^ degradation. A 1% w/v Cresyl Violet in 100% ethanol solution was prepared and kept at −20 °C. Brain sections were stained and dehydrated by immersion into the chilled Cresyl Violet-ethanol solution for ~20 s. Slides were air-dried and immediately use for LCM. We did not observe a negative impact on the ability of the laser to cut and release tissue samples dehydrated in this manner. To preserve NAD^+^ quality, it is important to process only one mouse sample at a time.

#### Laser-captured microdissection

Flat cap microcentrifuge tubes (Nippon Genetics, FG-021) were placed into the holder of the laser-captured microdissection microscope (Leica LMD 6000 system, Leica Microsystems, Buffalo Grove, IL, USA; Lever for 4 × 0.2 ml PCR-tube, 11505131). Thirty microliters of 10% HClO_4_ was pipetted into the cap. Sections were mounted and hypothalamic regions of interest were identified and cut. Collected samples were directly put into 10% HClO_4_. For normalization, cut areas were measured and saved. After collection, 90 μl HClO_4_ was added to a microcentrifuge tube to bring the sample volume to 120 μL. Samples were then briefly centrifuged to pool them in the bottom of the microcentrifuge tube. Samples may be stored at −80 °C at this stage.

### Animal studies

C57BL/6J male mice were purchased from Charles River Japan or Jackson Laboratory. Mice were maintained ad libitum on a regular chow diet on a 12 h light/dark cycle. All animal studies were reviewed and approved by the Institutional Animal Ethics Committees at RIKEN-BDR and the Institute of Biomedical Research and Innovation in the FBRI, Kobe, Japan (Figs. 1–4,6) or by the Institutional Animal Care and Use Committee of Washington University in St. Louis, Missouri, USA (Protocol No. 22-0007) (Fig. 5).

### NAD^+^ measurement by HPLC

NAD^+^ levels were determined using an HPLC system (Shimadzu) with a Supelco LC-18-T column (15 × 4.6 cm, Sigma)^21^. Briefly, LCM-collected samples or frozen tissues were extracted on ice with 10% perchloric acid for 15 min and centrifuged at 15,300 rpm for 5 min. The collected supernatant was neutralized by the addition of 3 M K_2_CO_3_ at a ratio of 1:3 (K_2_CO_3_:perchloric acid volume) for 15 min and then centrifuged at 15,300 rpm for 5 min. 5 M phosphate buffer was mixed with the neutralized supernatant at a 1:10 ratio and placed into an HPLC vial before loading into the HPLC system. The HPLC was run at a flow rate of 1 ml/min with 100% buffer A (50 mM phosphate buffer, pH 7.4) from 0–5 min, a linear gradient to 95% buffer A/5% buffer B (100% methanol) from 5–6 min, 95% buffer A/5% buffer B from 6–11 min, a linear gradient to 85% buffer A/15% buffer B from 11–13 min, 85% buffer A/15% buffer B from 13–23 min, and a linear gradient to 100% buffer A from 23–24 min.

### Quantitative real-time RT-PCR

Frozen brain sections cut at 25–30 μm were mounted on PEN-membrane slides (Leica) and kept on dry ice. The mounted slides were hydrated sequentially in 100, 95, 75, and 50% ethanol for 30 s each. The hydrated slides were stained with 1% Cresyl Violet (Sigma) for 1 min and dehydrated with 50, 75, 95%, and two cycles of 100% ethanol for 30 s each. The dehydrated slides were then incubated in xylene twice for 1 min each. After being air-dried for 5 min, the ARC, VMH, DMH, and LH were dissected by laser microdissection using the Leica LMD 6000 system (Leica Microsystems, Buffalo Grove, IL, USA)^34^. RNA was isolated from each hypothalamic nucleus using the Arcturus PicoPure RNA Isolation Kit (Life Technologies, Grand Island, NY, USA). Each RNA concentration was determined by NanoDrop, and cDNA was synthesized using the Applied Biosystems High-Capacity cDNA Reverse Transcription Kit (Life Technologies, Grand Island, NY, USA). qRT-PCR was conducted with the TaqMan Fast Universal PCR Master mix and appropriate TaqMan primers for each gene in the GeneAmp 7500 fast sequence detection system (Applied Biosystems). Relative expression levels were calculated for each gene by normalizing to GAPDH (glyceraldehyde 3-phosphate dehydrogenase) expression levels and then to a control.

### EV isolation and injection

Plasma collected from young mice (3–6 months old, female) was filtered with 0.22 μm filters (Millex-GP, Merck Millipore) pre-coated with 5% fatty acid-free BSA (Nakarai Tesque, Kyoto, Japan, 08587-84) in PBS. The filtered plasma was diluted in PBS and centrifuged at 210,000×*g* for 101 min at 4 °C (Beckman, Type 70.1Ti, Fixed angle ultracentrifuge rotor). EVs collected from three young mice were resuspended in 150 μl of PBS and administrated to 22-month-old female mice by intravenous (IV) injection. The IV injection was performed under inhalation anesthesia of 2% isoflurane (Pfizer Japan Inc., Tokyo, Japan) within 2 min. After 30 min, tissues were collected for NAD^+^ measurement.

### NMN/EV supplementation

For NMN injection experiments, aged mice (22 months) were intraperitoneally (IP) injected with NMN at a dose of 300 mg/kg. Controls were injected with PBS intraperitoneally. After 30 min, tissues were collected for NAD^+^ measurement.

### Statistical analyses

All data are presented as mean ± SE. Statistical significance between control and experimental samples was determined by unpaired Student’s *t*-test with statistical significance being *p* < 0.05.

## Data Availability

All data generated or analyzed during this study are available from the corresponding author on reasonable request.
